# Parent's preferences for unscheduled paediatric healthcare: A discrete choice experiment

**DOI:** 10.1111/hex.13802

**Published:** 2023-06-20

**Authors:** Emma Nicholson, Thérèse McDonnell, Ciara Conlon, Aoife De Brún, Edel Doherty, Eilish McAuliffe

**Affiliations:** ^1^ School of Psychology Dublin City University Dublin 9 Ireland; ^2^ UCD Centre for Interdisciplinary Research, Education and Innovation in Health Systems (IRIS), UCD School of Nursing, Midwifery and Health Systems UCD College of Health and Agricultural Sciences Dublin Ireland; ^3^ J.E. Cairnes School of Business & Economics National University of Ireland Galway Galway Ireland

**Keywords:** children, discrete choice experiment, unscheduled healthcare

## Abstract

**Background:**

Unscheduled healthcare is a key component of healthcare delivery and makes up a significant proportion of healthcare access, with children being particularly high users of unscheduled healthcare. Understanding the relative importance of factors that influence this behaviour and decision‐making is fundamental to ensuring the system is best designed to meet the needs of users and foster appropriate cost‐effective usage of health system resources.

**Objective:**

The aim of the study was to identify the parent's preferences for unscheduled healthcare for a common mild childhood illness.

**Design:**

A discrete choice experiment (DCE) was developed to identify the preferences of parents accessing unscheduled healthcare for their children.

**Setting and Participants:**

Data were collected from parents in Ireland (*N* = 458) to elicit preferences across five attributes: timeliness, appointment type, healthcare professional attended, telephone guidance before attending and cost.

**Results:**

Using a random parameters logit model, all attributes were statistically significant, cost (*β* = −5.064, 95% confidence interval, CI [−5.60, −4.53]), same‐day (*β* = 1.386, 95% CI [1.19, 1.58]) or next‐day access (*β* = 0.857, 95% CI [0.73, 0.98]), coupled with care by their own general practitioner (*β* = 0.748, 95% CI [0.61, 0.89]), identified as the strongest preferences of parents accessing unscheduled healthcare for their children.

**Discussion:**

The results have implications for policy development and implementation initiatives that seek to improve unscheduled health services as understanding how parents use these services can maximise their effectiveness.

**Patient or Public Contribution:**

The development of the DCE included a qualitative research component to ensure that the content accurately reflected parents experiences when seeking healthcare. Before data collection, a pilot test was carried out with the target population to gather their views on the survey.

## 1 INTRODUCTION

Unscheduled healthcare, which constitutes unplanned, nonroutine utilisation of health services, is a key component of healthcare delivery and makes up a significant proportion of healthcare access.^1^ Unscheduled healthcare is delivered mostly through general practitioners (GPs), out‐of‐hour GP services and emergency departments (EDs), as well as other services such as urgent care centres and minor injury units. There is an increasing demand for unscheduled services such as GP services^2^ and EDs.^3^ A survey of respondents in 34 countries found that 18%–40% of people surveyed had used an ED in the past year with lower ED use associated with greater accessibility of primary care.^4^ Indeed, the unavailability of appointments with the GP within a reasonable timeframe (e.g., within 24 h) has been found to cause parents to seek healthcare in the ED^5^ with the more flexible service offered by out‐of‐hours (OOH) care also leading patients to these services.^6^ Increased attendance at EDs and OOH services has implications for health service policy and planning that aims to provide adequate primary care services in their community and critically, impacts patient experience through overcrowding, longer waiting times and the increased costs of hospital care. However, understanding patients' needs and preferences when they first initiate contact with a health service is vital to foster more efficient and cost‐effective use of health services.

Patient preferences and other drivers of healthcare access must be accounted for to inform efficient, unscheduled care models that are responsive to patient needs and ensure patients use services in an intended manner.^7^ Discrete choice experiments (DCEs) are commonly used to identify patient preferences due to their ability to elicit rich data on patient decision‐making and preferences when accessing healthcare services.^8^ In DCEs, participants make a number of preference choices, which allows for quantifying tradeoffs between features of a particular health service,^8^ and this information can subsequently be used to inform healthcare policy and delivery. DCEs have been used to identify preferences for models of primary care with a multitude of preferences identified as influencing patient decision‐making when selecting primary care services^7^ as well as OOH GP care.^9^ However, unscheduled care is delivered across a number of components of the health system, and previous research has shown patients take all possible service options into consideration when making engaging in health‐seeking behaviour.^10^ With regard to the development of paediatric healthcare, first contact care is a key priority for child health services,^11^ and reducing demand for EDs, particularly in relation to visits that are deemed nonurgent, by directing patients to alternative services such as primary or urgent care services is critical.^10^

Children are particularly high users of unscheduled healthcare,^1^ and how parents navigate unscheduled healthcare depends on a multitude of complex, interrelated factors. In addition to this, children are an important group to examine in isolation as parents behave differently when seeking healthcare for their children and would be less likely to adopt a ‘wait and see’ approach, which is reflected in lower urgency presentations that may be treated in emergency and urgent services.^12^, ^13^ Moreover, the inability to access primary care in a timely manner diverts parents to seek care in higher acuity services such as EDs.^14^ Perceived urgency also influences patients to seek expedited care in EDs,^13^ particularly pertinent to parents, a population that consistently reports a need to minimise risk and seek reassurance.^15^ Children's conditions may deteriorate swiftly, generating additional anxiety when making decisions on behalf of their children, especially in the case of younger children, yet unable to communicate.^16^ A systematic review also identified GP–parent relationship, proximity to an ED, and perceived waiting times as influencing a parent's healthcare seeking reassurance.^17^

Patients will navigate a health system to best serve their needs and preferences at any given time and as a result, the features of the services available at any given time will influence where care is sought. The aim of the study was to identify the parent's preferences for unscheduled healthcare for a mild, self‐limiting illness. Thus, this study uses a DCE methodology that integrates attributes common across all services that offer unscheduled care to get a broader understanding of parent preferences when seeking unscheduled healthcare for their children.

## 2 METHODS

### 2.1 Data collection

The survey was administered online through Qualtrics™ in February 2021 and data from a random sample of 458 respondents was collected through Qualtrics^TM^ research panels. The objective of the sampling was to achieve a representative sample of parents in Ireland. Before completing the survey, all participants were asked to confirm they were over 18 years of age and parents of children living in Ireland. The DCE survey captured demographic information such as the parent's age, gender, medical card status, medical insurance and employment status. Data from eight participants were removed ahead of the analysis due to incorrect data (e.g., age of child greater than 18 years or the number of healthcare visits reporting as an unlikely amount [i.e., 2677]). The final analysis was conducted with the remaining 450 respondents.

### 2.2 Study design

DCEs are underpinned by random utility theory^18^ providing the respondent with several hypothetical choice alternatives, which are characterised by a number of attributes that differ in their levels across alternatives. Participants must make tradeoffs between attributes when deciding which alternative to choose, therefore, identifying the most important attributes in the respondent's decision‐making.^8^

### 2.3 Attribute selection

The attribute development process is critical to ensuring the DCE is unbiased, relevant and useful for policy making.^19^ A systematic review and qualitative research were conducted to generate the attributes that would populate the DCE. The systematic review synthesised studies (*n* = 56) examining factors that influence parental decision‐making when seeking unscheduled paediatric healthcare.^17^ For the qualitative component, semistructured interviews (*n* = 19) and one focus group (*n* = 4) were carried out with parents living in Ireland to understand parental health‐seeking behaviour for their children.^15^

When reviewing possible attributes generated through these methods, the research team adhered to guidelines as set out by^19^: first, that attributes should pertain to the commodity, that is, unscheduled care providers and not personal traits of the respondent, second, attributes should not be overly dominant and third, attributes should be important to the respondent. Following this process, five attributes were selected, as outlined in Table 1 below.

**Table 1:** DCE attributes and levels.

Attribute	Level
How long to wait for an appointment	Same day
	Next day
	Two days' time^a^
Appointment System	Appointment between 9:00 AM and 5:00 PM
	Appointment for any time including evening/weekend
	No given appointment but may have to wait for an unknown amount of time to be seen^a^
Advice before attending	No advice^a^
	Telephone advice from a healthcare professional about what to do
Who you will see	The practice nurse^a^
	Any doctor or nurse
	Your own GP
Cost	€0^a^
	€15
	€30
	€45

Abbreviations: DCE, discrete choice experiment; GP, general practitioner.

^a^ Reference category.

Attribute levels represent characteristics of services that offer unscheduled care. Dummy coding was used to code the levels in the categorical attributes. The levels of the cost attribute, which was included as a continuous variable, were set with reference to the cost of accessing primary and ED care in Ireland. As of 2017, 33% of the Irish population qualified for free access to general practice and public hospital care^20^ as holders of a General Medical Services (GMS) card. A further 10% qualified for free access to GP care as holders of a GP visit card. Entitlement to a GMS card is means tested or based on having a specified chronic illness, while entitlement to a GP visit card is also means tested with a higher income threshold. All children aged under six are also entitled to a GP visit card. Therefore, the lower bound for cost was set at no charge (€0). Those without a GMS or GP visit card pay an average of €51 per visit to their GP^21^ or an OOH service, €100 for an ED visit at a public hospital, and €75 to attend a local injury unit (LIU). As a substantial number of parents are unused to paying for medical care, the maximum cost was set at €45, with the remaining two cost options set at €15 increments.

### 2.4 Experimental design

**Figure 1:**
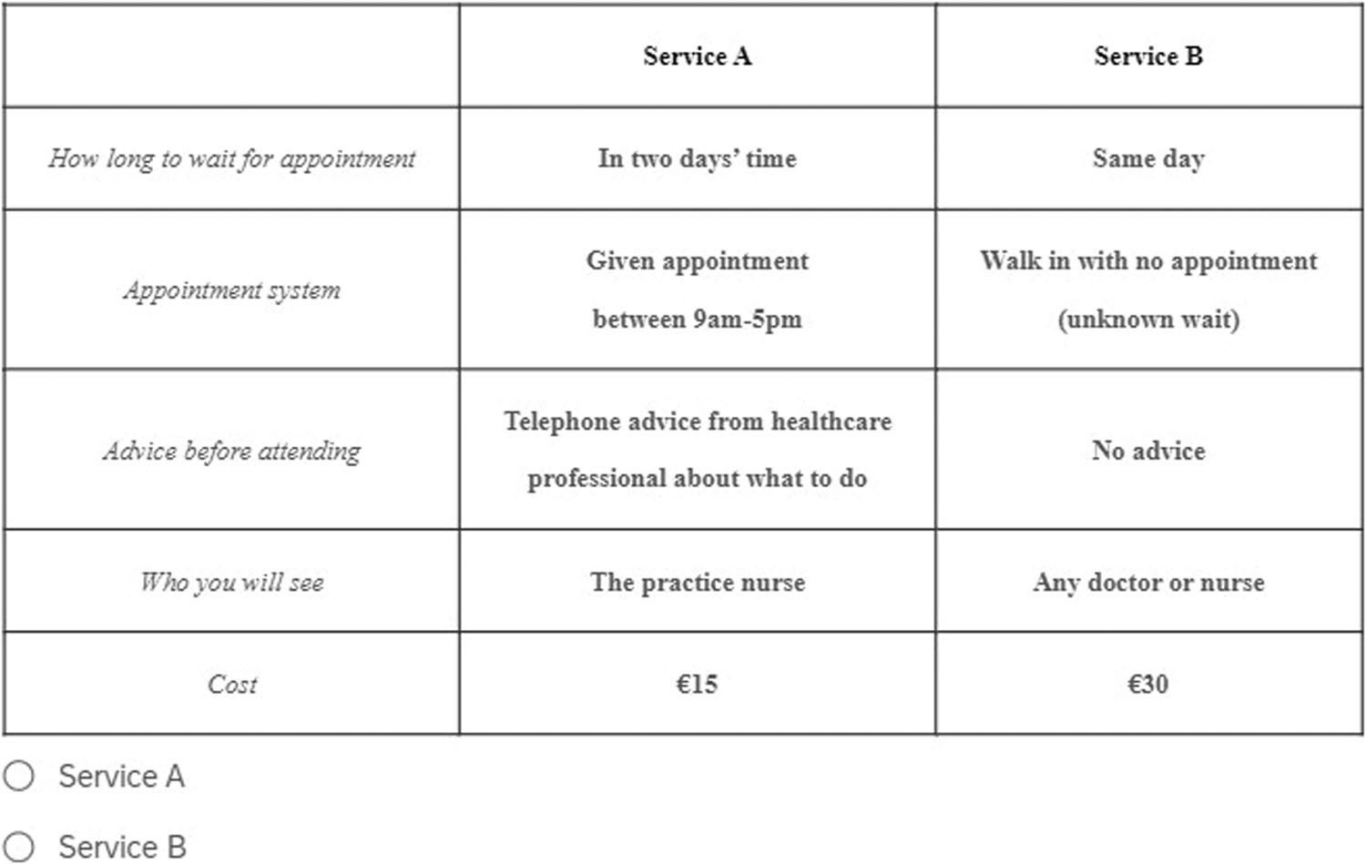
Example of a choice set in the discrete choice experiment survey.

Once the attributes and levels were selected, a Bayesian efficient design, based on minimising the Bayesian D‐error criterion, was used to develop the choice sets and the alternatives using Ngene^TM^ software. In total, 24 choice sets were created, and a blocked design split the choice sets into 2 blocks of 12 to minimise the burden on respondents. An example of a choice set used in the study is presented in Figure 1. Face validity was assessed before an initial pilot (*n* = 80) in January 2021, after which the design was updated to adjust the wording of the levels of one attribute (appointment type) and to update priors to generate the experimental design for the main sample.

The vignette presented the following scenario for all choice sets:

The next section will ask you to choose your preferred health service in a set of hypothetical scenarios. For each scenario, please imagine that your youngest (or only) child has not been well (not been themselves) for a period of time. You have managed the illness to the best of your ability; however, you have now decided that you need further support from a health professional in a health service [each attribute is then described as per Figure 1]. When responding to these scenarios, we would like you to think of a time before the COVID‐19 pandemic and to not consider how the pandemic may impact your choice

Each choice card contained two alternatives and the respondent chose their preferred option, service A or service B. Parents were presented with a forced choice, that is, there was no opt‐out option. A forced choice was included as the vignette was set up so that parents had already decided that, in their opinion, their child needed medical care. Given parents will have different thresholds for seeking care, we opted for the forced choice so as not to add this additional factor to the DCE.

The accessibility of local healthcare services (according to the participant), taking into account distance, transport and appointment availability, was also assessed on a scale of 1 (very easy) to 5 (very difficult). Questions were also asked about the health status and service utilisation of the respondent's youngest child, and all parents were asked to answer the choices presented with this child in mind. The youngest child was selected as younger children have higher rates of healthcare utilisation,^1^, ^22^ and it was assumed that asking parents to respond on each child would lengthen the survey and add unnecessary complexity.

### 2.5 Data analysis

The analysis was completed using Stata® 16. A conditional logit model with robust standard errors was first estimated to determine the general direction and significance of attributes and covariates on the choice of service. To further examine unobserved heterogeneity amongst respondents, random parameters mixed logit model was estimated firstly in preference space. Random parameter models estimated in the willingness‐to‐pay (WTP) space were also undertaken to estimate the WTP for the noncost attributes.^23^

A conditional logit model with robust standard errors was first estimated to determine the general direction and significance of attributes and covariates on the choice of service. This model assumes the respondents' utility (U) is determined as follows:

(1) $${U_{\mathrm{ij}}=\beta \mathrm{'X}}_{j}+\;{\gamma \mathrm{'Z}}_{i}+{ɛ}_{\mathrm{ij}},$$

where *i* refers to the respondent, and *j* each alternative presented as part of the choice set. β is estimated from a vector of attributes (X) describing the alternative (*j*), *Z* is a vector of individual characteristics that do not vary over alternatives but do vary over individuals and ${ɛ}_{\mathrm{ij}}$ is the stochastic disturbance representing unobserved characteristics of respondents. The utility gained from a chosen option must be higher than that of the alternative. In this study, each respondent was presented with 2 alternatives (*j*), service A and service B, and there were 12 choice sets for each respondent to answer. Respondent characteristics are alternatively invariant and only matter if they alter preferences. Therefore, interactions between attributes and family status, characteristics of the youngest child, mother's employment and educational status, and accessibility of health services, were assessed individually to identify variations in preferences.

To further examine unobserved heterogeneity amongst respondents, random parameters mixed logit model was estimated first in preference space. Each parent responding to the survey was presented with a number of scenarios (*s*) and was required to choose between two alternatives (*j*):

(2) $$U_{ijs}=\beta \prime_{i}X_{ijs}+\varepsilon_{ijs},$$

$X_{\mathrm{ijs}}$ is a vector of attributes of the healthcare service (see Table 1), $\beta_{i}^{\prime }$ is a vector of individual‐level coefficients, and ${ɛ}_{\mathrm{ijs}}$ captures the unobserved factors that influence choice. The noncost attributes are assumed to be normally distributed while the cost attribute is negative log normal. The simulation is based on 500 Halton draws.

Random parameter models estimated in WTP space were also undertaken to estimate the WTP for the noncost attributes. The marginal rate of substitution using WTP was also assessed in WTP space to estimate WTP^23^:

(3) $$\text{Marginal}\;\text{WTP}=MUx^{k}/-MUc,$$

where *MU* $x^{k}$ is the marginal utility of attribute $x^{k}$ and *MUc* is that of cost. The ratio of the noncost to cost coefficient is computed giving a direct WTP estimate. The noncost attributes were normally distributed and the cost coefficient incorporates differences in scale across respondents and is assumed to be random and log‐normally distributed with a negative distribution.

The Stata® user‐written packages *mixlogit* and *mixlogitwtp* were used to estimate the preference models and WTP mixed logit models respectively.^24^, ^25^

## 3 RESULTS

### 3.1 Participants

Table 2 presents the demographic characteristics of the respondents. Of the total sample (450), 65% were female and the mean age was 39.6 years (SD = 8 years). The mean number of children for respondents was 2 (SD = 1) and the mean age of the youngest child was 7 (SD = 4.5 years). Irish was the predominant ethnicity (71%), 68% were married and most had a minimum of third‐level education (58%). A higher proportion of parents were employed, with 51% working full‐time and 12% of respondents describing themselves as healthcare professional. A medical card was held by 39% of respondents, somewhat higher than the national average of 33%, with a further 12% holding a GP visit card. Half of the respondents had private health insurance, slightly higher than the national figure of 46.2%.^26^ When asked about the health status of their youngest child, 19% stated their child had an ongoing condition or disability, while 23% stated their child had a previous condition that required ongoing healthcare. The average number of healthcare attendances for the youngest child in the past year was 2.6 (SD = 7.2). When asked about the accessibility of their GP, OOHs GP and the ED on behalf of their youngest child, 9% assessed GP access as either difficult or somewhat difficult, compared with 22% for OOHs GP and 23% for ED. These results can be seen in Table 2.

**Table 2:** Descriptive statistics of the sample.

Variables (columns are % and *n*, unless otherwise stated)	Final sample (*N* = 448)	
Female	65%	294
Age of respondent (mean/SD)	39.6	8.1
Number of children (mean/SD)	2.2	1.2
Age of youngest child (mean/SD)	7.0	4.5
*Ethnicity*		
Irish	70%	317
Other White	17%	76
Other	13%	57
*Family status*		
Married	68%	305
Co‐habiting	14%	64
Divorced/separated/single/unknown	18%	81
*Highest level of education*		
Secondary	20%	89
Postsecondary	22%	98
Third level	58%	264
*Employment status*		
Working full‐time	51%	231
Working part‐time	21%	93
Stay‐at‐home parent	22%	98
Unemployed	6%	28
Healthcare professional	12%	56
*Medical card/GP visit card/Insurance*		
Medical card	39%	175
GP visit card	12%	52
Health insurance	50%	227
*Youngest child*		
Youngest child's health		
Very healthy, no problems	69%	309
Healthy, but a few minor problems	28%	126
Sometimes quite ill/almost always unwell	3%	15
Youngest child has conditions/disabilities		
Ongoing conditions or disabilities	19%	86
Previous conditions or disabilities, not ongoing currently	23%	105
Number of times accessed healthcare in past 12 months (mean/SD)	2.6	7.2
*Access to health service perceived to be somewhat difficult/difficult*		
GP	9%	39
OOH GP	22%	89
Emergency Department	23%	96

Abbreviations: GP, general practitioner; OOH, out of hours; SD, standard deviation

All attributes were significant in the conditional logit model and in the expected direction (see Table 3). The strongest factor was same‐day access (*β* = 0.935, 95% confidence interval, CI [0.796, 1.07]) which was followed by next‐day access (*β* = 0.609, 95% CI [0.516, 0.702]), being seen by your own GP (*β* = 0.502, 95% CI [0.392, 0.613]), an evening or weekend appointment (*β* = 0.305, 95% CI [0.221, 0.389]), a 9:00 AM to 5:00 PM appointment (*β* = 0.264, 95% CI [0.186, 0.341]), telephone advice (*β* = 0.237, 95% CI [0.177, 0.297]), being seen by any nurse or doctor (*β* = 0.152, 95% CI [0.072, 0.233]) and cost (*β* = −0.015, 95% CI [−0.018, −0.012]). Characteristics of respondents interacted with each attribute to determine the variation in preferences, and two interactions were statistically significant. Those stating that they perceived access to a GP to be *somewhat difficult* or *difficult* were less likely to prefer attending their own GP (*β* = −0.334, 95% CI [−0.545, −0.123]) and preference for a same day appointment was greater for those with more than one child (*β* = −0.283, 95% CI [−0.579, 0.013]). The positive and significant Alternative Specific Constant suggests that parents were considering other factors when making their decision.^27^

**Table 3:** Results from conditional logit model.

	Odds ratio	Robust standard error	CI 95%	*β*	Robust standard error	CI 95%
*Timeliness (base: 2 days' time)*						
Same day	2.548***	0.180	2.22, 2.93	0.935***	0.071	0.796, 1.07
Next day	1.839***	0.087	1.68, 2.02	0.609***	0.047	0.516, 0.702
*Appointment system (base: walk‐in)*						
Appointment 9:00 AM to 5 PM	1.301***	0.052	1.20, 1.41	0.264***	0.039	0.186, 0.341
Appoint available evenings and weekend	1.357***	0.058	1.25, 1.48	0.305***	0.043	0.221, 0.389
*Who patient will see* (*base: Practice nurse*)						
Any nurse or doctor	1.165***	0.048	1.04, 1.26	0.152***	0.041	0.072, 0.233
Own GP	1.653***	0.093	1.48, 1.85	0.502***	0.056	0.392, 0.613
Telephone advice available (base: No advice)	1.267***	0.039	1.19, 1.35	0.237***	0.031	0.177, 0.297
Cost (€0, €15, €30, €45)	0.985***	0.002	0.982, 0.988	−0.015***	0.001	−0.018, −0.012
Alternative Specific Constant	1.170***	0.042	1.09, 1.25	0.157***	0.035	0.087, 0.226
*N*	10,800					
Log likelihood	−3537					
AIC	7092					
BIC	7157					

*Note*: Standard errors are in parentheses.

Abbreviations: AIC, Akaike information criterion; BIC, Bayesian information criteria; CI, confidence interval; GP, general practitioner.

*** *p* < .001.

### 3.2 Random parameters logit model results

A random parameters logit model was estimated in preference space and all attributes and that were statistically significant in the conditional logit model remained significant in the preference model (Table 4), including the interactions (see Supporting Information: Table 1). The strongest factor was cost (*β* = −5.064, 95% CI [−5.60, −4.53]) followed by same‐day access (*β* = 1.386, 95% CI [1.19, 1.58]) next‐day access (*β* = 0.857, 95% CI [0.73, 0.98]), being seen by your own GP (*β* = 0.748, 95% CI [0.61, 0.89]), an evening or weekend appointment (*β* = 0.390, 95% CI [0.28, 0.50]), a 9:00 AM to 5:00 PM appointment (*β* = 0.363, 95% CI [0.25, 0.47]) and telephone advice (*β* = 0.312, 95% CI [0.22, 0.40]) and being seen by any nurse or doctor (*β* = 0.299, 95% CI [0.19, 0.41]). The standard deviation for all attributes, other than a preference for an appointment the next day and a consultation with any nurse or doctor, were statistically significant, indicating substantial heterogeneity in preferences. WTP in preference space was highest for an appointment on the same day (€27.36) followed by an appointment the next day (€16.93), compared with an appointment in 2 days' time. The next highest was an appointment with their own GP (€14.77), followed by the option of an evening or weekend appointment (€7.70) and an appointment between 9:00 AM and 5:00 PM (€7.16). Finally, respondents were willing to pay €6.16 for telephone advice and €5.90 to be seen by any nurse or GP in the practice. The cost coefficient was significant and in the expected direction suggesting parents preferred to pay less overall, and the statistical significance of the standard deviation suggests variability in this response.

**Table 4:** Results from random parameters logit models in both preference space and willingness to pay space.

Random parameters: Mean	Preference			Willingness to pay		
	***β***	Robust standard error	95% CI	***β***	Robust standard error	95% CI
*Timeliness (base: 2 days' time)*						
Same day	1.386***	0.099	1.19, 1.58	66.994***	5.421	56.37, 77.62
Next day	0.857***	0.063	0.73, 0.98	39.028***	3.704	31.77, 46.29
*Appointment system (base: walk‐in unknown wait time)*						
Appointment 9:00 AM to 5:00 PM	0.363***	0.055	0.25, 0.47	14.961***	3.321	8.45, 21.47
Appointments are available at times including evenings and weekend	0.390***	0.058	0.28, 0.50	14.980***	2.788	9.51, 20.45
*Seen by* (*base: Practice nurse*)						
Any nurse or doctor	0.299***	0.055	0.19, 0.41	8.784***	2.715	3.46, 14.10
Your own GP	0.748***	0.073	0.61, 0.89	28.811***	3.652	21.65, 35.97
Telephone advice available (base: no advice)	0.312***	0.045	0.22, 0.40	12.043**	1.786	8.54, 15.54
Cost (€0, €15, €30, €45)	−5.064***	0.274	−5.60, −4.53	−4.027***	0.112	−4.25, −3.81
Alternative Specific Constant	0.232***	0.042	0.148, 0.315	5.245**	2.224	0.89, 9.60
*Standard deviation of random parameters*						
Timeliness (base: 2 days' time)						
Same day	1.215***	0.093		67.489***	5.983	
Next day	0.253	0.143		23.059***	3.279	
Appointment system (base: walk‐in unknown wait time)						
Appointment 9:00 AM to 5:00 PM	0.288**	0.108		1.702	3.470	
Appoint available at times including evenings and weekend	0.278*	0.120		1.744	3.694	
Seen by (base: Practice nurse)						
Any nurse or doctor	0.082	0.194		1.800	4.423	
Own GP	0.700***	0.083		35.750***	4.041	
Telephone advice available (base: no advice)	0.370***	0.071		2.050	3.339	
Cost (€0, €15, €30, €45)	2.275***	0.247		1.064***	0.150	
Individual choices (*n*)	10,800			10,800		
Observations (*N*)	450			450		
Log likelihood	−3129			−3350		
AIC	6292			6735		
BIC	6416			6859		

*Note*: Standard errors are in parentheses.

Abbreviations: AIC, Akaike information criterion; BIC, Bayesian information criteria; CI, confidence interval; GP, general practitioner.

*** *p* < .001

** *p* < .01

* *p* < .05.

When estimated in the WTP space, the model fit was not as good as in the preference space. All attributes remained statistically significant, however, the interactions were not statistically significant in this model (see Supporting Information: Table 1). WTP was highest for a same‐day appointment (€66.99), followed by a next‐day appointment (€39.03) compared with an appointment in 2 days' time. Parents were willing to pay €28.81 to see their own GP versus the practice nurse but had a lower WTP to see any doctor or nurse (€8.78). Respondents were willing to pay for an appointment system versus walk‐in with an unknown wait time (€14.96 for 9:00 AM to 5:00 PM weekdays; €14.98 for a time including evenings and weekends), and €12.04 for telephone advice. As in preference space, the coefficient on cost was significant and in the expected direction.

## 4 DISCUSSION

This study utilised a DCE survey to assess parent's preferences when seeking first‐contact unscheduled healthcare for their children. While all attributes were significant in the model, the results suggest that same‐day or next‐day access, as well as being seen by their own GP (i.e., a GP they were familiar with), were the strongest preferences of parents accessing unscheduled healthcare for their child. Other attributes included an appointment during evenings and weekends, appointments during standard working hours, the option to be seen by any GP or practice nurse and telephone advice. The present findings enhance the current literature in this area by focusing exclusively on children and by considering all unscheduled health services as one entity rather than distinctive services to identify the common factors that influence parents' decision‐making.

Timeliness was the most important factor identified in the analysis as parents preferred to be seen on the same or the next day which reflects previous findings in the literature.^28^ Many parents access healthcare to seek reassurance that their child's illness is not serious or will not become more urgent. Therefore, once they have decided to seek healthcare, this study shows that timely access is the single most important attribute, with parents' preference strongest for same‐day or next‐day access. While certain clinical factors may lead a parent to select an ED or LIU over a GP, there are many presentations of an ill child that are suitable for care across all settings. Young children make up a large proportion of ED attendances that may have been treatable at primary care.^12^ Redirecting such presentations through primary care, leaving hospital resources available for those that need specialist diagnostics and care, has been a policy goal for many health systems internationally. However, increasing access to primary care is not guaranteed to reduce ED attendance in this population,^22^ and therefore, understanding the drivers of decision‐making and behaviour is critical. Parents will continue to utilise emergency and OOH services if they are more likely to offer same‐day care to balance their child's needs with other important responsibilities^13^ such as work commitments, particularly those with inflexible work arrangements,^6^ caring for other children and childcare requirements.

With the next strongest preference for attending their own GP, many parents would prefer same‐day access to their own GP to care at an ED or LIU, findings that are consistent with DCEs that focused on a particular type of healthcare.^28^ Younger children make up a large proportion of ED visits amongst paediatric patients^22^ and therefore, providing greater support to parents of younger children should be an important focus for policy and planning. Strengthening parents’ ability to cope with unexpected illness may reduce the utilisation of unscheduled services.^13^ For instance, first aid training and education for new parents could foster greater confidence in their capacity to recognise and handle common childhood illness (reference removed for peer review). Moreover, access to telephone support before an appointment was a preferred option for parents in the DCE and is offered by the majority of GP practices in Ireland.^29^ The use of remote consultations allowed for essential health services to continue during the COVID‐19 pandemic and evidence is continuing to emerge regarding the benefits and pitfalls of this approach.^30^ It is possible that this could provide a convenient,^6^ timely and cost‐effective approach to provide support and reassurance to parents of young children.

### 4.1 Limitations

A number of limitations of the study were identified. The DCE was designed to ensure it could be completed by parents without being cognitively challenging and could be completed within an acceptable timeframe. While the attribute development process identified the attributes and levels most relevant to parents' decision‐making on accessing unscheduled healthcare (references removed for peer review), other attributes, such as characteristics of the consultation and location of the health service, are also relevant and do not feature in this study. Moreover, it is important to note that the study is related to a mild illness which is common in childhood, however, the findings may not be relevant to presentations to unscheduled health services. Finally, data collection occurred during the COVID‐19 pandemic. While the vignette asked parents not to answer in the context of the pandemic, responses may have been impacted by the prevailing context and its impact on health‐seeking behaviour.^22^

## 5 CONCLUSIONS

Parents often navigate the health system as a single entity with many entry points. Understanding the relative importance of factors that influence this behaviour and decision‐making is fundamental to ensuring the system is best designed to meet the needs of users and foster the appropriate cost‐effective usage of health system resources. Timely same‐day access was a critical factor for parents when choosing unscheduled healthcare for their children, with care by their own GP as the second most important attribute. There is a need to recognise the factors that drive health‐seeking behaviour when engaging in policy development and implementation to improve service provision.

## AUTHOR CONTRIBUTIONS

**Emma Nicholson:** Conceptualization; investigation; methodology; formal analysis; project administration; writing—original draft. **Thérèse McDonnell:** Conceptualization; methodology; formal analysis; writing—original draft. **Ciara Conlon:** Investigation; methodology; project administration; writing—original draft. **Aoife De Brún:** Conceptualization; funding acquisition; methodology; writing—review and editing. **Edel Doherty:** Methodology; formal analysis; writing—review and editing. **Eilish McAuliffe:** Conceptualization; funding acquisition; methodology; writing—review and editing.

## CONFLICT OF INTEREST STATEMENT

The authors declare no conflict of interest.

## ETHICS STATEMENT

Ethical approval for the study was obtained from University College Dublin Human Subjects Ethical Review Committee (Ref: LS‐18‐107‐McAuliffe). Each participant provided informed consent on their own behalf before taking part in the study.

## Supporting information

Supporting information.

## Data Availability

The data that support the findings of this study are openly available in Zenodo at https://zenodo.org/record/6572717#.Y9kbd3bP24Q reference number [6572717].
