# Supplementary material for: Parent's preferences for unscheduled paediatric healthcare: A discrete choice experiment
Source: Health Expect. 2023 Jun 20;26(5):1931–40. doi: 10.1111/hex.13802 (PMC10485340; doi:10.1111/hex.13802)
Supplement: Supplementary file 1 — Supporting information. [file HEX-26--s001.docx]

| **Supplementary Table 1. Random Parameters Logit Models in Preference and WTP space with interactions** | | | | | | | | | |
| --- | --- | --- | --- | --- | --- | --- | --- | --- | --- |
|  | | Preference | |  | Willingness to Pay | | | | |
| **Random parameters: Mean** | | **ß** | **Robust Std Error** | **95% CI** | **ß** | **Robust**  **Std Error** | **95% CI** | |  |
| Same Day Appointment_One child | | -.589* | (0.159) | -.905, -.273 | -12.59 | 9.176 | -30.57, 5.40 | |  |
| GP Access problematic_Own GP | | -.443* | (.216) | -.867, -.019 | -20.57 | 10.58 | -41.32, .178 | |  |
|  | |  |  |  |  |  |  | |  |
| *Timeliness (base: 2 days’ time)* | |  |  |  |  |  |  | |  |
| Same day | | 1.577*** | (0.113) | 1.36, 1.79 | 67.089*** | (5.094) | 57.10, 77.07 | |  |
| Next day | | 0.858*** | (0.063) | .74, .98 | 38.855*** | (3.205) | 32.57, 45.14 | |  |
|  | |  |  |  |  |  |  | |  |
| *Appointment system (base: walk-in unknown wait time)* | | | |  |  |  |  | |  |
| Appointment 9am - 5pm | | 0.362*** | (0.054) | .25, .47 | 16.441*** | (3.110) | 10.34, 22.54 | |  |
| Appointment available at time including evenings and weekend | | 0.393*** | (0.058) | .28, .51 | 15.663*** | (2.634) | 10.50, 20.82 | |  |
|  | |  |  |  |  |  |  | |  |
| *Seen by (base: Practice nurse)* | |  |  |  |  |  |  | |  |
| Any nurse or doctor | | 0.298*** | (0.055) | .19, .41 | 8.999*** | (2.558) | 3.98, 14.01 | |  |
| Your own GP | | 0.781*** | (0.074) | .64, .93 | 31.771*** | (3.570) | 24.77, 38.77 | |  |
|  | |  |  |  |  |  |  | |  |
| *Telephone advice available (base: no advice)* | | 0.316*** | (0.045) | .23, .40 | 13.493*** | (1.714) | 10.13, 16.85 | |  |
|  | |  |  |  |  |  |  | |  |
| *Cost (€0, €15, €30, €45)* | | -5.097*** | (0.279) | -5.64, -4.55 | -3.977*** | (0.109) | -4.19, -3.76 | |  |
|  | |  |  |  |  |  |  | |  |
| *Alternative Specific Constant (ASC)* | | 0.231*** | (0.042) | .149, .314 | 5.630** | (2.261) | 1.19, 10.06 | |  |
|  | |  |  |  |  |  |  | |  |
| *Standard deviation of random parameters* | |  |  |  |  |  |  | |  |
| Same Day Appointment_One child | | .014 | .318 |  | 24.881** | 8.645 |  | |  |
| GP Access problematic_Own GP | | .513 | .414 |  | 17.976 | 16.612 |  | |  |
|  | |  |  |  |  |  |  | |  |
| *Timeliness (base: 2 days’ time)* | |  |  |  |  |  |  | |  |
| Same day | | 1.167*** | (0.088) |  | 62.463*** | (6.218) |  | |  |
| Next day | | 0.218 | (0.166) |  | 23.898*** | (3.035) |  | |  |
|  | |  |  |  |  |  |  | |  |
| *Appointment system (base: walk-in unknown wait time)* | | |  |  |  |  |  | |  |
| Appointment 9am - 5pm | | 0.262* | (0.113) |  | 3.438 | (2.725) |  | |  |
| Appoint available at time including evenings and weekend | | 0.286* | (0.119) |  | 1.239 | (2.881) |  | |  |
|  | |  |  |  |  |  |  | |  |
| *Seen by (base: Practice nurse)* | |  |  |  |  |  |  | |  |
| Any nurse or doctor | | 0.078 | (0.149) |  | 3.255 | (4.357) |  | |  |
| Own GP | | 0.651*** | (0.086) |  | 34.860*** | (4.356) |  | |  |
|  | |  |  |  |  |  |  | |  |
| *Telephone advice available (base: no advice)* | | 0.359*** | (0.071) |  | 9.671* | (3.109) |  | |  |
|  | |  |  |  |  |  |  | |  |
| *Cost (€0, €15, €30, €45)* | | 2.325*** | (0.247) |  | .995*** | (0.143) |  | |  |
|  | |  |  |  |  |  |  | |  |
| Individual choices (n) | | 10800 |  |  | 10,800 |  |  | |  |
| Observations (N) | | 450 |  |  | 450 |  |  | |  |
| Log-likelihood | | -3119 |  |  | -3347 |  |  | |  |
| AIC | | 6281 |  |  | 6736 |  |  | |  |
| BIC | | 6434 |  |  | 6889 |  |  | |  |
|  | |  |  |  |  |  |  | |  |
|  | *^Standard errors in parentheses. *** p<0.001, ** p<0.01, * p<0.05^* | | | | | | |  |  |
